# The Accumulation of Volatile Compounds and the Change in the Morphology of the Leaf Wax Cover Accompanied the “Anti-Aging” Effect in *Anethum graveolens* L. Plants Sprayed with 6-Benzylaminopurine

**DOI:** 10.3390/ijms242015137

**Published:** 2023-10-13

**Authors:** Anna V. Shirokova, Lev B. Dmitriev, Sergey L. Belopukhov, Valeria L. Dmitrieva, Irina L. Danilova, Viktor A. Kharchenko, Olga A. Pekhova, Elena F. Myagkih, Andrey N. Tsitsilin, Alexander A. Gulevich, Ekaterina V. Zhuravleva, Yulia N. Kostanchuk, Ekaterina N. Baranova

**Affiliations:** 1 Genetic and Cytology Laboratory, Federal State Budgetary Scientific Institution, Federal Scientific Vegetable Center (FSVC), Selektsionnaya 14, VNIISSOK Village, 143072 Moscow, Russia; 2 Department of Chemistry, Russian State Agrarian University—Moscow Agricultural Academy Named after K.A.Timiryazev (RSAU-MTAA), Timiryazevskaya 49, 127434 Moscow, Russia; lbdmitriev@yandex.ru (L.B.D.); sbelopuhov@rgau-msha.ru (S.L.B.); vl_dmitrieva64@mail.ru (V.L.D.); 3 Federal State Budgetary Scientific Institution, Research Institute of Agricultural of Crimea’, Kievskaya 150, 295493 Simferopol, Russia; oreol-mir@mail.ru (I.L.D.); olga.pehova@mail.ru (O.A.P.); origanum.science@mail.ru (E.F.M.); 4 Selection and Seed Poduction of Green Spice-Flavoring and Flower Crops Laboratory Federal State Budgetary Scientific Institution, Federal Scientific Vegetable Center (FSVC), Selektsionnaya 14, 143072 Moscow, Russia; kharchenkoviktor777@gmail.com; 5 Botanical Garden of All-Russian Research Institute of Medicinal and Aromatic Plants, Grina 7/1, 117216 Moscow, Russia; fitovit@gmail.com; 6 All-Russia Research Institute of Agricultural Biotechnology, Timiryazevskaya 42, 127550 Moscow, Russia; a_gulevich@mail.ru (A.A.G.); greenpro2007@rambler.ru (E.N.B.); 7 Federal State Budgetary Scientific Institution Belgorod Federal Agrarian Scientific Center of Russian Academy of Sciences, 308001 Belgorod, Russia; zhuravla@yandex.ru; 8 N.V. Tsitsin Main Botanical Garden of Russian Academy of Sciences, Botanicheskaya 4, 127276 Moscow, Russia

**Keywords:** exogenous cytokinin, dill, essential oil, volatile compounds, wax cover, fine structure

## Abstract

Essential oils (EOs) are of commercial importance for medicine, food, cosmetics, the perfume industry, and agriculture. In plants, EOs, like the wax cover, serve as protection against abiotic stresses, such as high temperatures and water deficiency. The use of spraying with exogenous hormones of aromatic plants affects the accumulation and composition of volatile compounds, as well as tolerance to abiotic stress. As a result of cytokinin treatment with 6-BAP (6-benzylaminopurine) (200 mg L^−l^) of *Anetum graveolens* L. “Uzory” and “Rusich” varieties, several responses to its action were revealed: a change in the division of leaf blades, inhibition of flowering, an increase in the content of EO and its main components *α*-phellandrene and *p*-cymene in leaves, and limonene in umbels and fruits. It was revealed that the increased accumulation of EO in dill leaves was longer with sufficient moisture. In contrast, under conditions of heat and water deficiency, the effect of 6-BAP treatment on accumulations of the EO in leaves was short-lived and did not appear on umbels and fruits. The study of the cytokinin effect on a fine structure of a wax cover on the adaxial side of leaves by scanning electron microscopy revealed a change in its elements (from amorphous layers with scales to thin tubules), which probably increased the sensitivity of leaves to water deficiency and, consequently, led to a decrease in the biosynthetic activity of leaf tissue. Thus, 6-BAP had an impact on the adaptive properties of dill plants, prolonging the “youth” of vegetative organs and the ability to EO biosynthesis under conditions of sufficient moisture.

## 1. Introduction

One common way to increase EO levels is by spraying plants with natural hormones or synthetic growth regulator preparations [1,2,3]. The exogenous application of cytokinins to aromatic crops is used to improve the growth and development of leaves and inflorescences, increase the accumulation of essential oils, and change the component composition, as well as increase the resistance of these plants to abiotic stress factors [4,5].

Cytokinins, which are derivatives of adenine, are synthesized in plants mainly by cells of the root meristem and move acropetally to shoots, but they can also be formed in young leaves and even in fruits [6,7,8,9].

EOs have many useful properties; therefore, they are valuable for the food industry (as flavors and natural food preservatives), agriculture as an insecticide, and are also widely used in cosmetic product production and perfumery [10,11]. Their use in pharmacology and medicine occupies a special place since the main compounds of essential oils have a wide range of biological activity [12,13,14,15]. EOs are very complex natural highly concentrated hydrophobic liquid mixtures of plant secondary metabolites [16]. Their volatile components, as a rule, have an intense odor and have various terpenes, aldehydes, alcohols, ketones, and simple phenols [17]. The chemical composition of EOs may vary depending on the variety, organ and stage of plant development, and environmental conditions [18,19]. Terpenoids, or isoprenoids, which form the basis of EOs, are derivatives of isopentenyl diphosphate, and their biosynthesis occurs either through the mevalonic acid pathway (MVA pathway) from acetyl-CoA or through the non-mevalonic acid pathway or methyl erythritol phosphate (MEP), resulting in geranyl pyrophosphate (GPP), which is a precursor of monoterpenes [20].

Also, EOs provide plant protection against biotic and abiotic stresses, acting, for example, as antioxidants and antibiotics [21,22,23]. In addition, volatile isoprenoids are able to protect plants from thermal damage and allow them to maintain the rate of photosynthesis [24].

Terpenoids, together with other cyclic compounds (sterols and flavonoids), are also a part of epicuticular waxes that protect plants from water and heat stress. The cuticle and wax coating cover the entire outer surface of most terrestrial plants [25]. Features of micromorphology and the chemical composition of epicuticular wax contribute to the formation of a multifunctional barrier that protects plants from uncontrolled water loss, reflects excess solar radiation, and affects surface wettability. Typical vegetable waxes are complex mixtures of aliphatic compounds, consisting primarily of very long-chain fatty acids (VLCFA) and their oxygen functional derivatives, which are predominantly alcohols, ketones, aldehydes or esters of fatty acids, and cyclic components [26,27]. The characteristic ultrastructure of epicuticular wax crystals depends on the predominant class of compounds in these complex mixtures [28].

One of the sources of valuable essential oils with a wide range of applications is dill (*Anethum graveolens* L., family *Apiaceae*), a fragrance herb, whose greens and seeds have been used fresh or dried for a long time to flavor dishes and drinks, and are in the preparation of pickles, food and feed preservation, and other agricultural applications [29].

Major components of dill herb are *α*- and *β*-phellandrenes, *p*-cymene, *3,9-oxy-p*-menth-1-ene (dill ether), and *3,9-epoxy-p*-menth-1-ene, and in fruits, depending on the variety, these are *d*-limonene, carvone, dihydrocarvone, dillapiol [30,31,32,33,34]. Although *α*-phellandrene is a very common cyclic monoterpene found in the EO of many types of medicinal and aromatic plants, the herb *Anethum graveolens* is one of the best sources, with a high content (50 to 70%) combined with easy extraction.

Essential oil from the herb and fruits (“seeds”) of dill, as well as its individual pure components, and are used in various areas of medicine [35,36,37,38]. For example, *α*-phellandrene, limonene, and carvone have anticancer effects, while high biological activity is combined with low toxicity [39,40,41]. At the same time, *α*-phellandrene has antinociceptive, larvicidal, and insecticidal biological activities [42]. Like limonene, it has high antimicrobial activities against both antibiotic-susceptible and antibiotic-resistant bacteria, mainly via its ability to promote cell rupture and inhibition of protein and DNA synthesis [43]. There is also the prospect of using pure α-phellandrene against diseases associated with inflammation and elevated cytokines since it suppresses the overproduction of cytokines of IL-6 and TNF-α (suppressing the overproduction of pro-inflammatory cytokines of IL-6 and TNF-α) [44,45]. *D*-limonene promotes the death of bacteria resistant to synthetic antibiotics; for example, in association with gentamicin, its antibacterial activity against gram-positive and gram-negative bacteria has been clinically proven [46]. In addition, limonene is used clinically to dissolve gallstones containing cholesterol [47]. Carvone has anti-inflammatory and antidiabetic action on cellular and molecular targets that contribute to the induction of apoptosis, autophagy, and senescence [48]; causes relaxation of smooth muscles (its action is similar to a classical calcium channel blocker) [49]; and is promising for the creation of new antiallergic drugs [50]. *P*-cymene is an important aromatic compound used for the synthesis of fine chemicals for the production of flavoring compounds, herbicides, pharmaceuticals, and *p*-cresol [51,52,53]. Thus, in addition to the industrial value of a dill EO, its individual components are of value for the production of new-generation drugs.

Drought stress induces the formation of terpenoids in aromatic and spicy plants, which makes it possible to increase plant tolerance to elevated temperatures. It is also known that under the influence of ABA, an antagonist of cytokinins in various processes of plant growth and development [54,55,56,57], the biosynthesis of cuticular wax increases [58].

The purpose of this study was to reveal the “rejuvenating” effect of exogenous cytokinin *6-BAP* on the growth and development of *Anethum graveolens* plants, on the yield and component composition of the essential oil of leaves and generative organs, and on plant tolerance to drought stress conditions, which are related with this change in the wax coating on the surface of plant tissues.

## 2. Results

### 2.1. Effect of 6-BAP on Development and Morphological Traits of Dill Plants

**Figure 1:**
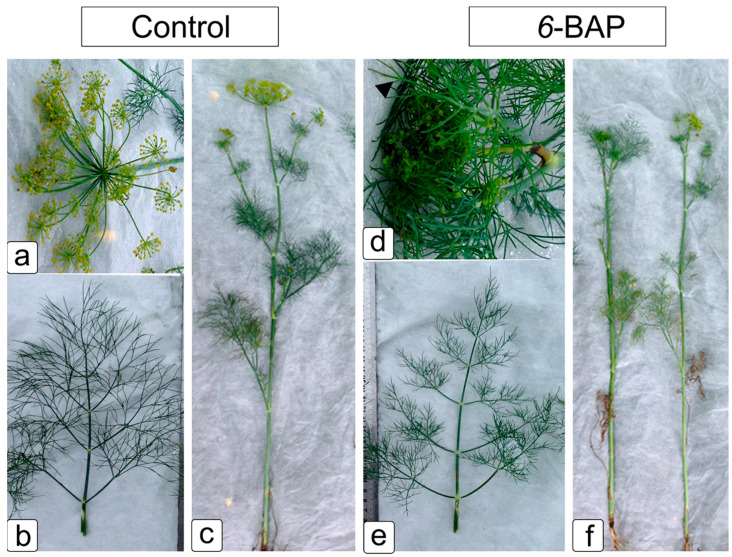
6-BAP effect on the growth and development of cv. “Uzory” dill (*Anethum graveolens* L.) plants. (**a**–**c**) Control, non-treated plants; (**d**–**f**) 6-BAP sprayed plants 23 days after treatment. Designations: (**a**,**d**)—umbels; (**b**,**e**)—leaves; (**c**,**f**)—plants. Bar: 5 cm.

Spraying with cytokinin caused a change in morphological traits in dill plants (Figure 1 and Figure S1). Control untreated plants cv. “Uzory” remained bright green (Figure 1c), while plants treated with *6*-BAP had lighter stem and leaf color (Figure 1f). The rachises of the leaves of the main stem became more branched compared to the untreated control, and the number of “third order” lobes increased from two to four (Figure 1b). Leaf segments have become shorter and thinner, similar to needles. The angle of the segments to the axes of the rachis changed. Leaf segments cv. “Rusich” became 1.5–2 times longer—up to 30 mm long and wider—up to 1.2–1.6 mm (for untreated plants—0.9–1.2 mm) (Figure S1b,e). In *6*-BAP-treated plants of both varieties, flowering of the terminal inflorescence came later by 5–7 days, and the lateral inflorescence by 8–10 days, compared with the control untreated plants. Visually, there was a faster wilting and further drying of the leaves of the treated plants compared to the control (Figure S1c,f).

The glaucous waxy shade was preserved on the control plants’ leaves, while in 6-BAP-sprayed plants, the leaf color became bright green, with a subtle waxy coating. The glaucous waxy tint was preserved on the control plants’ leaves, while in *6*-BAP-sprayed plants, the leaf color became bright green, with a subtle waxy coating.

**Figure 2:**
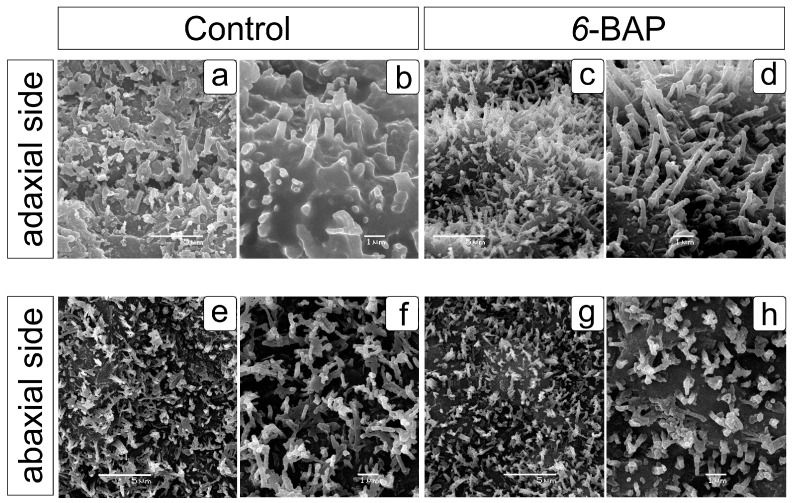
Arrangement of wax crystalloid forms on the leaf surfaces of control and *6*-BAP-treated cv. “Rusich” dill (*Anethum graveolens* L.) plants (22 DAT). Scanning electron micrographs: wax on the adaxial (**a**–**d**) and abaxial (**e**–**h**) sides of the leaf segments in the control (**a**,**b**,**e**,**f**) and *6*-BAP-treated (**c**,**d**,**g**,**h**) dill plants.

Scanning electron microscopy showed differences in wax formations on the leaf surfaces of the control and treated cv. “Rusich” dill plants (Figure 2). On the adaxial side of leaf segments of the control plants, the wax coating looked like an amorphous layer with short tubules (Figure 2b), while on the abaxial side, the surface also looked like a dense layer with densely branched rosettes and sparse tubules 0.6–1.8 µm long (Figure 2f). *6*-BAP-treated plants also had numerous rosettes and flakes on the abaxial side Figure 2g,h), and very densely arranged fine tubules (2.0–3.2 µm) on the adaxial side (Figure 2d), resembling pubescence. 

**Figure 3:**
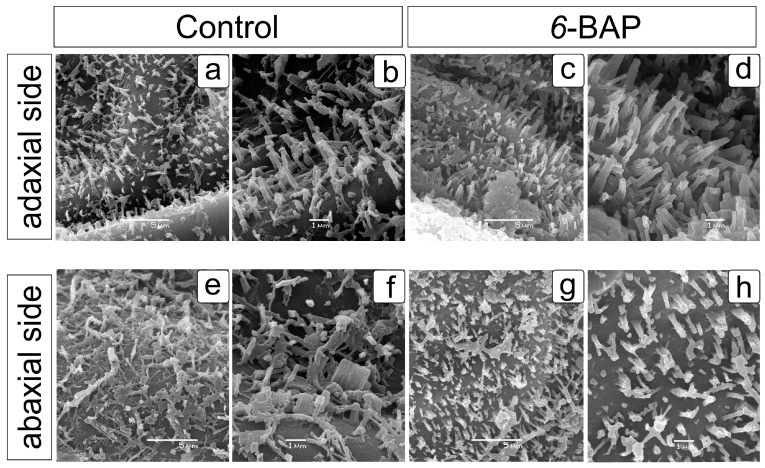
Arrangement of wax crystalloid forms on the leaf surfaces of the control and *6*-BAP-treated cv. “Uzory” dill (*Anethum graveolens* L.) plants (22 DAT). Scanning electron micrographs: wax on the adaxial (**a**–**d**) and abaxial (**e**–**h**) sides of the leaf segments in the control (**a**,**b**,**e**,**f**) and *6*-BAP-treated (**c**,**d**,**g**,**h**) dill plants.

There are no contrasting differences between the architecture on the adaxial surface of leaf segments in the control (Figure 3a,b) and BAP-treated (Figure 3c,d) plants of cv. “Uzory”. In both cases, the waxy structures are tubes. In BAP-treated plants, the height of these structures is about 1.7–2.3 µm, while in the control plants, they are 1.6–2.1 µm. On the abaxial side, these structures are also similar and represent short tubes and clumps of wax (Figure 3a–d) [59]. It is possible that the adhesion is caused by the lower stability of the elements than on the adaxial side and the effect of pre-treatment during the preparation of the samples.

### 2.2. Yield of EO and Its Component Composition

**Figure 4:**
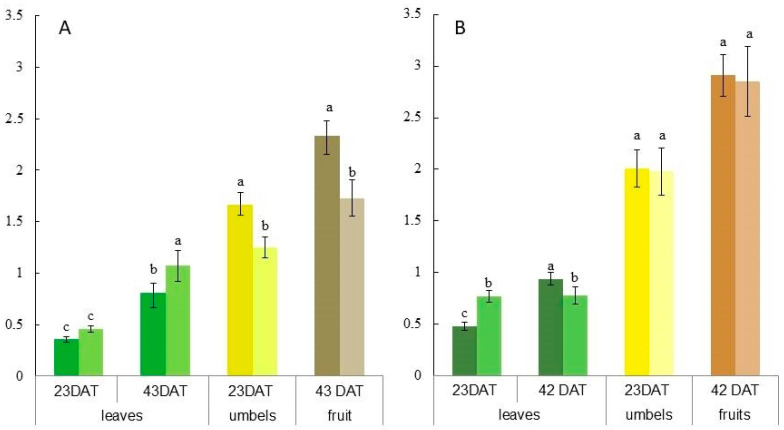
EO content (g/100 g) in leaves, flowering umbels, and fruits of dill plants of cv. ”Uzory” (**A**) and cv. “Rusich” (**B**) after treatment with *6*-BAP (200 mg L^−1^). Designations: dark colors are the samples from control plants, light colors are the samples from plants after 6-BAP spraying. The letters a, b, c mean *p* ≤ 0.01.

Dill varieties differed in the content of EO in leaves, inflorescences, and fruits. In all organs, the EO amount was higher in cv. “Rusich” (Figure 4A,B). EO content in the leaves of the control untreated plants was the highest in both varieties at the beginning of seed maturation. Most of the EO was obtained from fruits in the milky stage (about 2.3 g/100 g dw in cv. “Uzory” and about 3 g/100 g dw in cv. “Rusich”). The content of EO in flowering umbels in cv. “Uzory” was about five times higher than in leaves, and in cv. “Rusich” the difference was four times. The content of EO in the leaves of treated plants cv. “Uzory” increased by two times during the transition from the flowering phase (23 DAT) to the fruiting phase (42 DAT), and reached 1 g/100 g dw, which was 20% higher than in the control plants at the same time. In *6*-BAP-treated cv. “Rusich” dill plants, the maximum content of EO in the leaves was obtained at 23 DAT and almost did not change at 42 DAT. Both in inflorescences and seeds, the EO content in the treated and control plants of cv. “Uzory” differed significantly, by 30%, while in cv. “Rusich”, the differences were insignificant.

The sum of the identified components in the EO of the studied varieties was more than 90%. In total, 45 components were identified in the essential oil from the leaves of “Uzory”, 46 components were identified in cv. “Rusich”, and 20 compounds were found in the seeds of both varieties (Table S1). It was found that *α*-Phellandrene, *3,9*-*Epoxy*-*p*-menth-1-ene, *p*-Cymene, and *β*-Phellandrene prevailed in the leaves and inflorescences of both varieties, and only two main compounds, carvone and *d*-limonene, were revealed in seeds, and the high content of *d*-limonene was also characteristic of inflorescences (Table 1). The content of almost all main components in untreated leaves of cv. “Uzory” dill plants increased significantly during plant development and their transition from the flowering stage to the fruiting stage. In the leaves of cv. “Rusich”, a significant increase (2.5 times) of the *p*-Cymene content was found, while the content of *3,9*-*Epoxy*-*p*-menth-1-ene decreased by one third. The same trend was noted in the treated dill plants: the content of *3,9*-*Epoxy*-*p*-menth-1-ene decreased by more than two times and the content of *p*-Cymene increased by more than four times, from 23 to 42 DAT. The amount of the main EO component contained in the green mass of dill, *α*-Phellandrene and in the leaves of treated cv. “Uzory” plants, was almost 1/2 higher in the flowering stage and 2/5 higher after 20 days. The content of *p*-Cymene in the treated and control untreated plants also significantly differed by 23 DAT: almost three times in cv. “Uzory” and two times in cv. “Rusich”. The amount of *β*-Phellandrene per 23 DAT was more than twice as high in 6-BAP-treated cv. “Uzory” plants compared to the untreated plants.

Significant fluctuations in the amount and ratio of the main compounds were also noted in inflorescences between *α*-Phellandrene and *D*-Limonene. In the control plants of both varieties, the content of D-Limonene in inflorescences exceeded the content of *α*-Phellandrene. In cv. “Rusich”, it is three times; after treatment, it is almost twice, and in cv. “Uzory”, the content of *α*-Phellandrene was 50% higher. In both cultivars, a twofold increase in *p*-Cymene content was also observed in flowering umbels after exposure to *6*-BAP (Table 1).

The content of limonene and carvone in the fruits of the control untreated plants of both varieties was approximately the same—about 45%. In the fruits of *6*-BAP-treated plants, there is about a quarter more limonene and two times less carvone than in the untreated plants. The content of D-limonene, the main compound in the fruits of the treated plants of both varieties, was approximately the same and was 2.4–2.7 times higher than carvone.

## 3. Discussion

Cytokinins are involved in processes such as cell division, shoot initiation and growth, delay in senescence and photomorphogenic development, control of chloroplast division and growth, regulation of metabolism and morphogenesis in response to environmental stimuli, and protection from abiotic stress [9].

As a result of the impact of 6-BAP on dill plants, the growth of stems and the formation of umbrellas, the onset of flowering phases, and the development of fruits slowed down (Figure 1), and in drought conditions, this slowdown was less noticeable than under normal humidity and did not affect fruit ripening. Plant generative development is known to be largely dependent on the gibberellin (GA) signal. GAs and cytokinins act antagonistically in controlling most developmental processes, including leaf formation and meristem maintenance [60,61]. In cv. “Rusich”, the change in leaf color from glaucous to bright green, caused by a change in the structure of the wax on the leaf surface, from a denser structure to a more refined variant of tubule arrangement, indicates possible biochemical changes in the composition of the wax (Figure 2). Earlier, in several studies, it was shown that the predominance of one of the components or a class of substances in the composition of epicuticular waxes determines the characteristic morphology of its elements. Thus, in various poplar species, the smooth surface of the wax coating on leaves without structural elements contains all five classes of wax substances with a predominance of long-chain FAs or alkanes [62,63]. Wax tubules of dill can be attributed to the type dominated by secondary alcohols [28]. Wax morphology is influenced by temperature, humidity, and light intensity, so it is difficult to assess the degree of influence of any individual component and associate it with a specific appearance. Thus, a change in the structure of the wax from tubules to more branched elements on the leaves of *Brassica oleracea* caused an increase in temperature during cultivation [64]. In *Arabidopsis thaliana*, cuticular wax biosynthesis was shown to decrease under dark conditions [65] and be induced by ABA or drought through an increased expression of *KCS2* [58]. On the contrary, the expression of the regulatory gene *CER2* in *Eceriferum* mutants with disturbances of waxy coating is not induced by light, temperature, drought, or other stress conditions, but can be induced by young leaf cytokinins [66]. In our research, the effect of cytokinin apparently affected the sensitivity of plants to an increase in temperature, which prevented the transformation of tubules on the adaxial leaf surface into branched structures. The change in the configuration of the components of the wax cover and the increase in the content of EO were noted at the same time—23 days after treatment with *6*-BAP. Plant volatile compounds, including terpenes, short-chain aldehydes, ketones, alcohols, and esters, can help “deliver” the wax to the cuticle surface, and the volatiles can remain in the boundary layer long enough to promote crystallization and interconversion of the wax structures [63].

An increase in the EO content in leaves of treated dill plants and a decrease in its content in inflorescences and fruits under normal moisture conditions clearly demonstrated that “rejuvenation” enhanced the secretion of EO in the leaves. Endogenous production, in combination with exogenous cytokinin, inhibited the generative development of plants, slowing down the accumulation of EO in the inflorescences and fruits of dill cv. “Uzory”. On the contrary, drought nullified the effect of cytokinin on dill, causing degradation of the hormone since drought stress leads to early leaf senescence, and among other things, causes the destruction of auxins and cytokinins [67]. Perhaps that is why the influence of cytokinin during drought was shorter, as evidenced by the almost equal accumulation of EOs in the inflorescences and fruits of the control and experimental plants of cv. “Rusich”.

In addition, it is possible that during hot and dry weather, the rejuvenating effect of *6*-BAP, which caused a change in the wax coating, reduced its protective effect on the leaves. As a result, the secretory ability of the leaves and the accumulation of EOs decreased. Thus, after treatment of *Brassica napus* plants with exogenous methyl jasmonate (MeJA) and salicylic acid (SA), an increase in the content of secondary alcohol in the wax was revealed, which caused a change in cuticle permeability and an increase in water loss by leaves [68].

Although more than 40 different components were found and identified in the studied varieties in the composition of EOs, only six compounds in the leaves and two in the fruits prevailed among them, accounting for more than 10% of the total. In our study, the content of the components characteristic of the leaves (*α*- and *β*-phellandrenes) in the treated plants was significantly higher than in the control, not only in the leaves of both varieties but also in the inflorescences of cv. “Rusich” plants.

**Figure 5:**
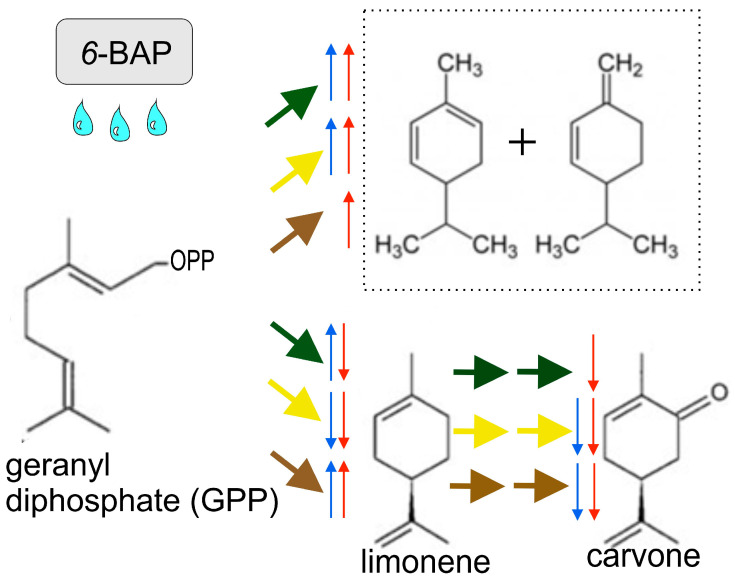
Scheme of changes for the synthesis of the main volatile components in the leaves, umbels, and fruits of dill after the application of *6*-BAP. Designation wide arrows: green—leaves, yellow—umbels, brown—fruits; thin arrows: blue indicates changes in synthesis in the organs of cv. “Uzory”; red—cv. “Rusich”.

It is possible that the enhanced accumulation of individual EO components in the treated plants compared to the control ones was associated with the prolongation of the development stage during which typical EO components are formed. In addition, an increased content of individual components in the EO may indicate that cytokinins specifically affect their biosynthesis (Figure 5). At the same time, the content of 3,9-*Epoxy*-*p*-menth-1-ene in the leaves of both varieties was lower than in the control plants, which was also shown in other studies. Thus, foliar spraying with cytokinins increased the content of almost all components of the EO, except for dill ether and dill apiol [5]. Similar results were obtained by Özel et al. when comparing the composition of EO from dried and fresh dill leaves [69]. These differences were even more pronounced in *Mentha longifolia*, which had limonene (40.8%) as the main compound in oven-dried leaves, whereas its fresh leaves were rich in pulegone (35%) [70].

The content of carvone was associated with both the variety and the stage of fruit development. The pathway for the conversion of limonene to carvone, described in detail for cumin fruits, is similar to both dill [53] and *Mentha* species in the leaves of which it is formed [71,72]. In cumin in the early stages of fruit development 5–10 days after pollination (DAP) due to the high activity of limonene synthase and *trans*-carveol-dehydrogenase, abundant limonene accumulates, but almost no carvone is formed due to a trace level of limonene-6-hydroxylase activity—the next enzyme in the chain of transformations. The accumulation of a significant amount of carvone per 10 DAP coincides with an increase in the activity of limonene-6-hydroxylase. It is important that the limonene in the developing cumin fruits, and hence the dill that has fallen into the EO ducts, can no longer turn into carvone. In cumin, this occurs between 15 and 20 DAP [73], and in dill, perhaps later. Thus, the protracted phase of inactive limonene-*6*-hydroxylase, probably caused by *6*-BAP, even at a high limonene content compared to the control, caused a low carvone content in the dill fruits of both varieties.

## 4. Materials and Methods

### 4.1. Plant Material

The following cultivars of *Anethum graveolens* L. bred at the Federal Scientific Center of Vegetable Production were used in this study.

Cultivar “Uzory”—a middle-late cultivar (60 days after seedling to flowering and 75 days after seed maturation). The leaves are green. The weight of 1000 seeds is 1.5 g. Plant height during the flowering stage is 130–145 cm. Cultivar “Rusich”—a middle-late cultivar (65 days after seedling to flowering and 80 days after seed maturation). The leaves are large and glaucous, and the ultimate segments are flattened. The weight of 1000 seeds is 1.8 g. Plant height during the flowering stage is 150–165 cm.

The plants were grown in the experimental field of the Federal Scientific Center of Vegetable Production (FSCVP) (west of Moscow Region) using the standard technique: there were four-line ribbons, the distance between ribbons was 20 cm, and the distance between plants in a line was 10–15 cm.

### 4.2. Spraying of Dill Plants with 6-BAP

At the bolting stage (3 stem leaves), dill plants were sprayed once with 100 mL of a freshly prepared solution of 6-benzylaminopurine (6-BAP) (200 mg L^−1^; the treated area was 8 m^2^). The treatment was performed in the evening, at 20 °C. The effect of 6-BAP on growth, flowering, and fruit formation in the dill plants was evaluated.

### 4.3. Essential Oil Extraction

Essential oil (EO) content was determined by hydrodistillation. Following treatment, the plant material was harvested twice: at 23 and 43 days after treatment (DAT) for cv. “Uzory” and 23 and 42 DAT for cv. “Rusich”, as well as from untreated (control) plants. Dill plants were cut above the third leaf (from the stem base). Leaves were separated from dried plants, whole umbels with flowers were cut off, young fruits were separated from umbel rays, and the material was dried to brittleness at 23–25 °C. The EO was extracted using a Ginsberg collector, and the components were identified by GC-MS. Dill fruits were separated from umbel rays before EO extraction. The material was ground and placed in a 1000 mL round-bottom flask; then, distilled water was added (1:2), and distillation was carried out for 90 min (leaves, flowers) or 120 min (fruits). The obtained EO yield was collected into the vials and weighed, and was calculated in m_eo_/100 g of plant material using the following formula:

$$\frac{(m_{vial\;with\;EO}-m_{empty\;vial})\times 100g}{sample\;weight}$$

$$m_{EO}/100g=\frac{(m_{vial\;with\;EO}-m_{empty\;vial})\times 100g}{sample\;weight}$$

Hydrodistillation was performed in triplicate. The results were presented as the mean value of the individual measurements with the corresponding standard deviation (SD) using Microsoft Office Excel software (Microsoft Inc., Redmond, WA, USA).

### 4.4. Essential Oil Component Identification by Gas Chromatography

Chromatographic identification of the essential oil components was performed using a GC-MS Clarus 600C (Perkin-Elmer, Rodgau, Germany) system at the Educational and Scientific Core Facilities Centre (Department of Chemistry, Moscow Timiryazev Agricultural Academy, Moscow). Chromatographic conditions: Elite-WAX capillary column (60 m × 0.32 mm × 0.25 µm); carrier gas—helium, 1 mL/min; sample volume—0.5 µL; split ratio 1:50; temperature program: 60 °C—5 min, 3°/min up to 195 °C, isotherm 15 min; FID (flame ionization) and MS (mass-spectrometry) detectors (simultaneously); and mode: E+ (70 eV), interface temperature—210 °C, and source temperature—180 °C. Essential oil components were detected by MS using the NIST/ERA/NIH mass spectral library (ver. 2-2005) to identify all compounds. The final results were obtained using the RI (chromatographic retention indices) library, developed at the Department of Physical and Organic Chemistry of the Moscow Timiryazev Agricultural Academy, Moscow, and based on the elliptical distribution of *n*-alkanes under arbitrarily selected temperatures of analysis. The concentrations of essential oil components are presented as a percentage of the sum of all components.

### 4.5. Scanning Electron Microscopy

To assess wax layer density, scanning electron microscopy (SEM) was used. Fresh leaves (seventh leaf from three treated and three control untreated plants) were cut 22 days after 6-BAP treatment (DAT). The leaves were immersed in 2.5% (*v*/*v*) glutaraldehyde (GA) in phosphate-buffered saline (PBS) at pH 7.2 for 3 days and then washed twice in phosphate-buffered saline, changing the buffer every washing. After that, the leaves were sequentially immersed into 50% and 96% (*v*/*v*) alcohol solutions and stored in 96% alcohol. Before SEM, the leaf segments were passed to fresh absolute ethanol to ensure the complete removal of water and were subjected to critical point drying on a dryer (Hitachi, Ibaraki, Japan). The leaf fragments were then mounted on stubs with double-sided adhesive carbon strips (Double Sided Carbon Tape, 8 × 20 mm, Shanghai, China). The samples were then coated with gold using an Eiko IB-3 Ion Coater (Eiko Engineering Co., Tokyo, Japan). The thickness of the coating layer was 20 nm. The adaxial and abaxial leaf surfaces were examined using a JSM-6380LA scanning electron microscope (JEOL Technics Ltd., Tokyo, Japan) at 20 kV accelerating voltage. The length of the wax “crystals” was determined using SEM Control User Interface software Version 7.11 (JEOL Technics Ltd., Tokyo, Japan). The wax structure was described using the terminology proposed by Barthlott et al. (1998) [59].

## 5. Conclusions

Cytokinin *6*-BAP caused a slowdown in the development of dill plants, probably enhancing the synthesis of those substances that are inherent in a certain stage of plant development (vegetative). Under normal moisture conditions, the effect of “rejuvenation” was longer than under conditions of water deficit and heat. In combination with heat stress and drought, exogenous treatment with the hormone caused a dramatic increase in the biosynthesis of EO and its main components in the leaves but, apparently, due to a decrease in wax protection associated with a change in the structure of the wax coating, a decrease in secretory ability subsequently occurred. On the contrary, *6*-BAP had no noticeable effect on the accumulation of EO in flowers and fruits under these conditions but prevented the conversion of limonene to carvone.

For practical purposes, late varieties of dill with a long germination to fruiting period can serve as a source of phellandrenes and limonene, and the use of *6*-BAP, especially in conditions of sufficient moisture, can increase their content.

## Figures and Tables

**Table 1:** Main components of EO in leaves, inflorescences, and fruits of cv. “Uzory” and cv. “Rusich” dill plants at 23 and 42 (43) days after treatment with *6*-BAP (200 mg L^−1^).

	“Uzory”								“Rusich”							
EO Components	Leaves				Umbel		Fruit		Leaves				Umbel		Fruit	
	Control	BAP	Control	BAP	Control	BAP	Control	BAP	Control	BAP	Control	BAP	Control	BAP	Control	BAP
DAT	23		42		23		42		23		43		23		43	
*α*-Phellandrene	20.1 ^c^	32.5 ^b^	28.3 ^b^	37.3 ^a^	26.4 ^b^	38.2 ^a^	t	0.1	53.7 ^b^	68.5 ^a^	54.1 ^b^	69.2 ^a^	18.0 ^b^	25.1 ^a^	1.4 ^b^	2.4 ^a^
*D*-Limonene	5.4 ^b^	7.3 ^a^	9.3 ^a^	8.4 ^a^	34.9 ^a^	22.1 ^b^	49.7 ^b^	65.2 ^a^	6. 8 ^a^	5.6 ^b^	7.1 ^a^	6.2 ^b^	57.6 ^a^	46.8 ^b^	44.4 ^b^	67.1 ^a^
*β*-Phellandrene	4.1 ^c^	8.8 ^a^	10.1 ^a^	8.6 ^a^	4.4 ^b^	6.6 ^a^	t	0.1	8.3 ^b^	9.1 ^a^	9.3 ^a^	9.6 ^a^	2.7 ^b^	3.7 ^a^	0.3 ^b^	0.4 ^a^
*p*-Cymene	6.2 ^c^	17.7 ^b^	22.6 ^a^	16.2 ^b^	5.77 ^b^	12.0 ^a^	0.1 ^b^	0.3 ^a^	5.1 ^b^	2.2 ^c^	12.8 ^a^	8.4 ^b^	2.1 ^b^	4.1 ^a^	0.2 ^b^	0.4 ^a^
*3,9*-*Epoxy-p*-menth-1-ene	14.5 ^b^	8.2 ^c^	17.8 ^a^	9.9 ^b^	13.0 ^a^	11.9 ^a^	t	t	17.0 ^a^	10.7 ^b^	11.0 ^b^	4.6 ^c^	5.9 ^b^	9.9 ^a^	t	t
Carvone	0.6 ^a^	0.3 ^c^	0.8 ^a^	0.3 ^c^	9.3 ^c^	0.4 ^d^	46.3 ^a^	27.5 ^b^	0.7 ^a^	0.4 ^c^	0.2 ^c^	0.1 ^c^	9.8 ^a^	4.4 ^b^	48.0 ^a^	26.6 ^b^

t-trace ≤ 0.05%; values followed by the same letter within a line are not significantly different for each medium, according to Tukey’s test at *p* ≤ 0.05; that is, a > b > c > d. that is, a > b > c > d.

## Data Availability

The data are provided in the article.

## Supplementary Materials

The supporting information can be download at: https://www.mdpi.com/article/10.3390/ijms242015137/s1.
