# Supplementary material for: The Accumulation of Volatile Compounds and the Change in the Morphology of the Leaf Wax Cover Accompanied the “Anti-Aging” Effect in Anethum graveolens L. Plants Sprayed with 6-Benzylaminopurine
Source: Int J Mol Sci. 2023 Oct 13;24(20):15137. doi: 10.3390/ijms242015137 (PMC10606700; doi:10.3390/ijms242015137)
Supplement: Supplementary file 1 [file ijms-24-15137-s001.zip › ijms-2615228-supplementary.pdf]

Supplementary Materials

Table S1. Composition of EO components from leaves and umbels of dill (*Anethum graveolens* L.) cv. ‘Uzory’ and cv. ‘Rusich’.

	EO components	Area, %			
		cv. ‘Uzory’		*cv. ‘Rusich’*	
		leaves	umbels	leaves	umbels
1	α-Pinene	0,95	0,93	1,37	0,38
2	β-Thujene	0,10	0,16	0,10	0,02
3	Toluene	0,30	0,06	0,06	0,01
4	Camphene	0,02	0,02	0,04	0,01
5	β-Pinene	0,06	0,05	0,10	0,02
6	Sabinen	0,11	0,08	0,19	0,07
7	β-Myrcene	0,67	0,57	0,77	0,65
8	α-Phellandrene	28,30	26,39	54,09	18,04
9	D-Limonene	9,30	34,87	7,09	57,64
10	β-Phellandrene	10,13	4,39	9,31	2,69
11	1,3,8-p- Menthatriene	0,02	0,06	0,02	0,08
12	trans-β-Ocimene	0,16	0,01	0,03	0,08
13	*γ*-Terpinene	0,03	0,02	0,06	0,02
14	*p*-Cymene	22,56	5,77	12,81	2,10
15	Terpinolene	0,09	0,07	0,15	0,05
16	*D*-Fenchone	0,20	0,02	0,01	0,01
17	cis-Limonene 1,2-epoxide	0,01	0,02	0,01	0,02
18	p-Cymenene	0,10	0,02	0,06	0,02
19	trans-Limonene 1,2-epoxide	0,02	0,01	0,13	0,02
20	3,9-Epoxy-p-menth-1-ene	17,79	13,04	11,04	5,90
21	Carvenone	0,02	0,02	0,01	0,04
22	exo-Isocamphanone	0,13	0,04	0,03	t
23	4-Terpinenyl acetate	0,14	0,07	t	t
24	Bornyl acetate	0,09	0,01	0,05	0,01
25	Terpinen-4-ol	0,14	0,02	0,05	0,07
26	cis-Dihydrocarvone	0,09	0,21	0,01	0,25
27	trans-Dihydrocarvone	0,13	0,25	0,04	0,09
28	trans-Piperitol	0,30	0,02	0,12	t
29	Cryptone	0,32	0,05	0,17	0,02
30	Carvotanacetone	0,31	0,03	1,07	0,05
31	β-Cubebene	0,33	0,15	0,08	0,23
32	Phellandral	0,14	0,08	0,03	0,13
33	Carvone	0,84	9,33	0,23	9,81
34	Carveol	0,61	0,03	0,06	0,03
35	Sabinyl acetate	0,91	0,49	0,03	0,09
36	p-Mentha-2,8-dien-1-ol, cis-	1,49	0,31	0,01	0,07
37	cis-Sabinol	0,39	0,07	0,03	0,01
38	Thymol	0,19	0,15	0,01	0,02
39	Carvacrol	0,47	0,29	0,01	0,03
40	Myristicin	0,59	0,04	0,03	0,11

t-traces, <0,01% Note: some components, mainly sesquiterpenes and their derivatives, the content of which was less than 0.01%, were difficult to identify (not unambiguous) and are not listed in the table.

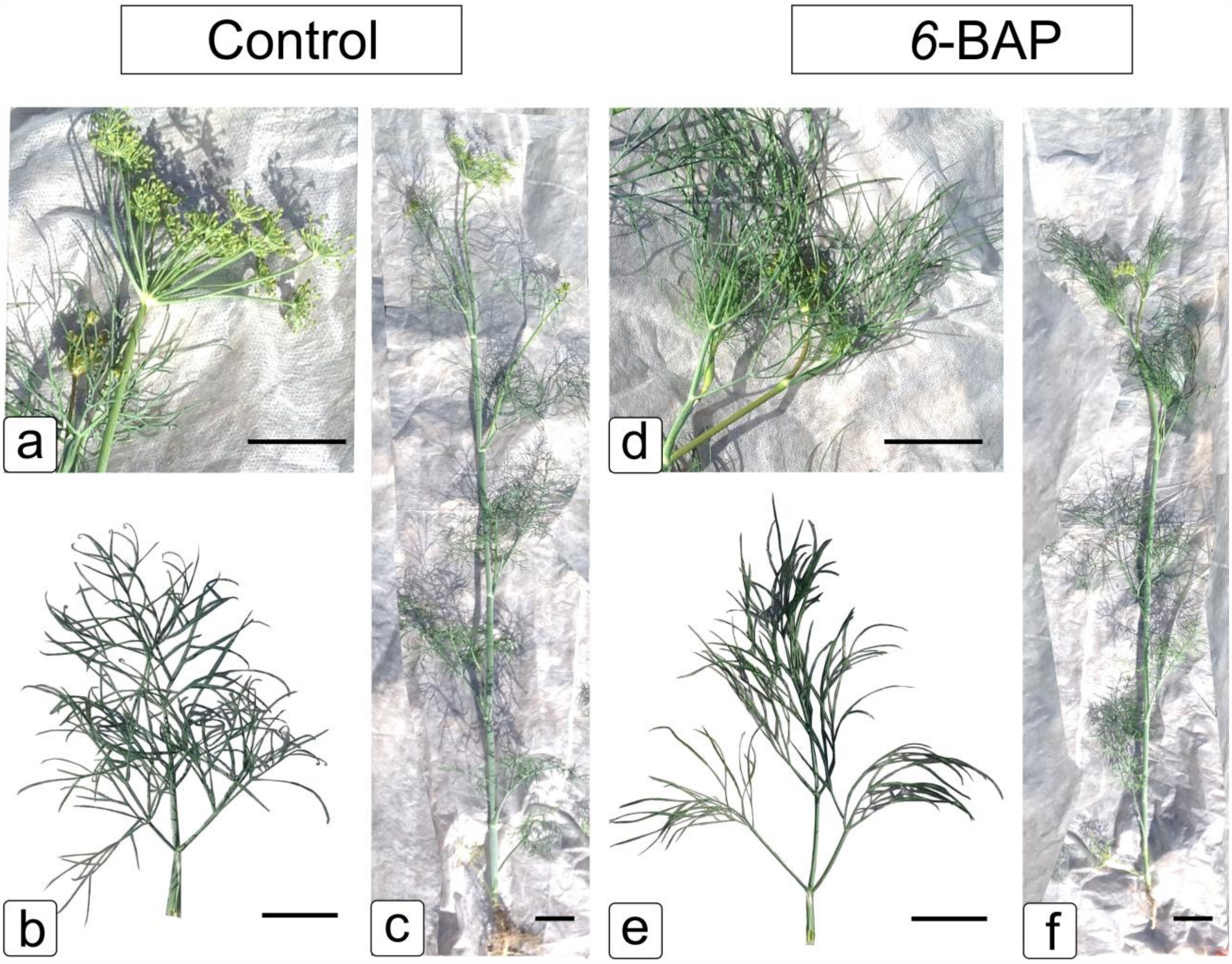

Figure S1. 6-BAP effect on growth and development of cv. ‘Rusich’ dill (*Anethum graveolens* L.) plants. (a-c) Control, not treated plants; (d-f) 6-BAP sprayed plants 23 days after treatment. Designations: (a, d) - umbels; (b, e) - leaves; (c, f) – flowering plants. Bar 5 cm.
